# A Review of the Antiviral Role of Green Tea Catechins

**DOI:** 10.3390/molecules22081337

**Published:** 2017-08-12

**Authors:** Jun Xu, Zhao Xu, Wenming Zheng

**Affiliations:** College of Life Sciences, Henan Agricultural University, 95 Wenhua Road, Zhengzhou 450002, China; zhaoxufight@163.com (Z.X.); zhengwenmingw@hotmail.com (W.Z.)

**Keywords:** green tea catechins, chronic infectious diseases, antiviral activity, mechanism

## Abstract

Over the centuries, infectious diseases caused by viruses have seriously threatened human health globally. Viruses are responsible not only for acute infections but also many chronic infectious diseases. To prevent diseases caused by viruses, the discovery of effective antiviral drugs, in addition to vaccine development, is important. Green tea catechins (GTCs) are polyphenolic compounds from the leaves of *Camellia* *sinensis*. In recent decades, GTCs have been reported to provide various health benefits against numerous diseases. Studies have shown that GTCs, especially epigallocatechin-3-gallate (EGCG), have antiviral effects against diverse viruses. The aim of this review is to summarize the developments regarding the antiviral activities of GTCs, to discuss the mechanisms underlying these effects and to offer suggestions for future research directions and perspectives on the antiviral effects of EGCG.

## 1. Introduction

**Figure 1:**
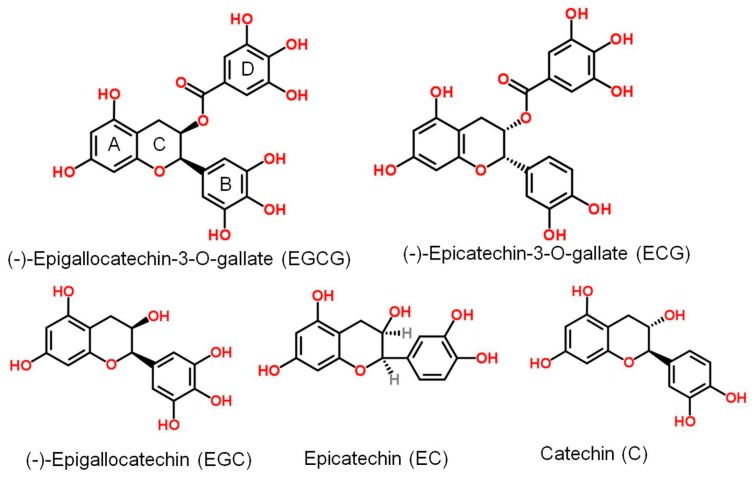
Structures of green tea catechins.

Plant-derived natural products assist in the prevention and treatment of various diseases. Green tea and its major constituent polyphenols (also known as green tea catechins, GTCs) are well known for their contributions to human health, including their antitumor, antioxidative, and antimicrobial activities [1,2]. Additionally, for thousands of years, green tea has been one of the most commonly consumed health-promoting beverages in many parts of the world [3]. Epigallocatechin-3-gallate (EGCG), one of the five types of catechins and a major component of GTCs, accounts for approximately 59% of the total polyphenols in dry green tea leaves (Figure 1). Several other components are epicatechin gallate (ECG), epigallocatechin (EGC), epicatechin (EC) and catechin (C). ECCG is generally considered the main active constituent of green tea.

Many types of infection diseases caused by various viruses have a considerably negative effect on human health and lives. Viruses are responsible for not only acute infections, but also many chronic infectious diseases. Hepatitis B virus (HBV) and hepatitis C virus (HCV) play significant roles in chronic hepatitis, liver cirrhosis and liver carcinoma [4]. The Epstein-Barr virus (EBV) can cause a chronic active infection characterized by chronic or recurrent infectious mononucleosis-like symptoms, stomach cancer, lung cancer, nasopharyngeal carcinoma, multiple sclerosis, and even systemic multi-organ involvement [5]. The human immunodeficiency virus (HIV) can invade vital cells in the human immune system and cause the progressive failure of the immune system named acquired immunodeficiency syndrome (AIDS) [6]. The original cause of chronic viral infectious diseases is the invasion by the infectious virus, therefore, effective antiviral treatments are very important. In recent years, GTCs have demonstrated inhibitory activities against various viruses, such as human viruses [7,8,9,10,11], livestock viruses [12,13], fish viruses, and even some arboviruses, such as dengue viruses (DENV)[14], Chikungunya virus (CHIKV) [15] and Zika virus (ZIKV) [16]. This review first systematically summarizes our current understanding of the antiviral effects of GTCs (Table 1) then and provides a perspective on EGCG and future research directions for the compound.

## 2. Inhibitory Effects of GTC on DNA Virus

### 2.1. GTC Inhibits HBV

Infection with hepatitis viruses is the main cause of hepatitis in the world. There are five types of hepatitis viruses: types A, B, C, D and E. Types B and C are the major cause of chronic liver diseases, such as liver fibrosis, cirrhosis and hepatocellular carcinoma. HBV is a serious threat to international human public health, as hundreds of millions of people are infected. Despite vaccination programs, the possibility of and potential for outbreaks and epidemic spread cannot be excluded. Unfortunately, the most commonly used medications or therapies for HBV have success rates that are dependent on viral genotype and suffer from several limiting side effects [17]. Therefore, finding and identifying more effective therapies without side effects is of great importance. 

HBV is a member of the *Hepadnavirus* family with a 3.2-kb genome of partially double-stranded DNA. In 2008, we reported the anti-HBV activity of GTCs in HepG2-N10, a stable cell line expressing HBV antigens [9]. We found that the expression of HBV antigen and the synthesis of extracellular HBV DNA, intracellular replicative intermediates, and cDNA and HBV mRNA were inhibited when HepG2-N10 cells were treated with various concentrations of green tea extracts. Other research groups have also documented similar results; for example, EGCG had strong effects on the expression of two HBV antigens and could prevent the production of HBV genomic DNA [18,19]. Using HepG2.117, an inducible HBV-replicating cell line, He et al. observed that EGCG significantly inhibited the RNA synthesis of intracellular replicative intermediates [20]. However, in HepG2.117 cells, the synthesis of HBV pregenome RNA, precore mRNA and HBeAg was not affected [20]. To clarify the molecular mechanism of the anti-HBV effects of EGCG, we used florescence quenching and affinity binding experiments [21]. We found that among five different GTCs, EGCG showed the strongest inhibition of HBV antigen expression. The associated mechanism may involve EGCG acting as an antagonist of the farnesoid X receptor alpha (FXRα) and the interaction between EGCG and FXRα down regulating the transcriptional activities of the HBV EnhII/core promoter [21]. 

In 2014, Huang et al. found that different genotypes of HBV could be inhibited by EGCG in immortalized human primary hepatocytes and two constructed cell lines, DMSO-differentiated HuS-E/2 cells and HA-NTCP-expressing Huh7 cells (NTCP is the receptor of HBV) [22]. Furthermore, in the membrane, clathrin-dependent endocytosis of NTCP was induced and directed to protein degradation pathways by EGCG. However, EGCG did not change HBV structures or the expression of genes involved in HBV entry. Recent evidence indicates that during infection, host cells can trigger autophagy, a lysosomal degradation mechanism that is important for cell survival. The results of Zhong and co-workers indicated that to combat the incomplete autophagy induced by HBV, EGCG can create a microenvironment that is detrimental to HBV replication by altering lysosomal acidification [23]. 

### 2.2. Effect of GTCs on Herpes Simplex Virus

Herpes simplex is a viral skin disease caused by infection with herpes simplex virus type 1 (HSV-1) or herpes simplex virus type 2 (HSV-2). Both HSV-1 and HSV-2 are enveloped viruses possessing a relatively large double-stranded, linear DNA genome and belong to the *Herpesvirus* family. HSV-1 is commonly spread through mouth-to-mouth contact and causes cold sores and genital herpes. HSV-2 is usually spread by sexual contact and generally causes only genital herpes [68]. The anti-HSV activity of GTCs was observed by Lyu et al. in 2005 [11]. The authors found that among the 18 tested flavonoids, EC, ECG, EGC and EGCG showed strong anti-HSV activity [11]. A subsequent investigation demonstrated that EGCG showed stronger activity against HSV than the other GTCs tested and made infectious clinical isolates of HSV-1 and HSV-2 lose their infection ability [24]. The data also showed that inactivation of the virus occurred because of a direct destructive effect of EGCG on the HSV-1 virions [24]. Another study from this research group showed that digallate dimers of EGCG could inactivate HSV and could be developed into more effective antiviral drugs against HSV [25]. The results of case studies from Zhao and co-workers suggested that a topical formulation containing EGCG-stearate in 100% glycerin could prevent and treat HSV-induced symptoms [26]. Palmitoyl-EGCG (p-EGCG), a modified EGCG, increased the effectiveness of EGCG as an anti-HSV agent in HSV-infected Vero cells [27]. An interesting study exploring the reason for the broad-spectrum antiviral activity of EGCG demonstrated that EGCG competitively interacted with virion surface proteins to inhibit the attachment of HSV-1 to heparan sulfate [28]. Moreover, in this study, EGCG showed its broad-spectrum antiviral activities on many other viruses, including HCV, IAV, murine cytomegalovirus (mCMV), vaccinia virus (VACV), vesicular stomatitis virus (VSV), reovirus, and adenovirus. This activity was possibly related to a common mechanism: the interaction between the virus and heparan sulfate or sialic acid was inhibited [28]. 

### 2.3. Effect of GTCs on the EBV

The EBV is another member of the herpes family and infects humans [69,70]. EBV is one of causes of many types of malignant tumors and certain autoimmune diseases. It produces lytic infections in most epithelial cells and latent infections in most B-cells (from which it reactivates periodically producing a reactivating infection) [71]. Some researchers investigated the anti-EBV effects of EGCG using different cell models. They found that EGCG not only suppressed the synthesis of some lytic proteins of EBV but also inhibited the lytic infection by downregulating the transcription of immediate-early genes or reducing the DNA binding potency of nuclear antigen [29,30]. Additionally, Liu et al. explored the molecular mechanisms of the anti-EBV activity of EGCG in spontaneous lytic infection in vitro [31]. The results showed that the anti-EBV lytic infection mechanisms of EGCG could be associated with inhibition of the MEK/ERK1/2 and PI3-K/Akt signaling pathways [31].

### 2.4. Effect of GTCs on Adenovirus

Adenovirus is an icosahedral non-enveloped DNA virus approximately 60–90 nm in diameter. Adenovirus infection can cause cold-like symptoms, sore throat, bronchitis, pneumonia and pink eye. People can contract an adenovirus infection at any age. Weber et al. found that EGCG at micromolar concentrations reduced the virus titers of adenovirus in two cell infection models and inactivated purified adenovirions [32]. The results from Colpitts and co-workers showed that EGCG inhibited the attachment of adenovirus by interacting with virion surface proteins [28]. 

## 3. Inhibitory Effects of GTCs on RNA Virus

### 3.1. GTC Inhibits HIV

In 1994, Chang et al. reported for the first time the anti-HIV activities of polyphenolic catechins from Chinese green tea [34]. The authors isolated EGCG, EC and ECG from *Camellia sinensis* and demonstrated their potential as new inhibitors of HIV reverse transcriptase (RT). HIV, the pathogen of AIDS, was identified by Barré-Sinoussi and Luc Montagnier in 1983 [72]. There are two types of HIV: HIV-1 and HIV-2. HIV-1 was initially discovered and is the predominant virus, while HIV-2 is less transmissible. There are around 1~2 million cases of HIV-2 infection with unique geographical and age distribution. According to the World Health Organization (WHO), since the beginning of the HIV epidemic, approximately 70 million people have been infected with HIV and more than 35 million people have died of various diseases caused by HIV. At the end of 2015, there were approximately 36.7 million HIV carriers globally [73]. Therefore, to cure AIDS, studying novel drugs and seeking new treatments is very important. Before major inroads had been made in anti HIV therapy and vaccine in the last few years [74,75], more than a dozen research groups paid efforts into the fields of anti-HIV effects of tea catechins, mainly anti-HIV-1. 

EGCG is an inhibitor of HIV reverse transcriptase. Chang et al. have reported that three catechins, namely, EC, ECG and EGCG, demonstrate strong inhibitory action against HIVRT [34]. Kinetic analysis showed that the tested catechins were competitive inhibitors of the template-primer and noncompetitive inhibitors of dTTP [34]. In human peripheral blood mononuclear cells (PBMCs), EGCG acting as an inhibitor of HIVRT could decrease the expression of the HIV p24 antigen, which subsequently resulted in inhibition of RT activity [8]. A report from Li et al. revealed that both HIV-1 and HIV-2 infection were suppressed by lower physiological concentrations of EGCG [35].

EGCG inhibits HIV entry into target cells. In 2002, Yamaguchi et al. reported that EGCG has a destructive effect on viral particles of HIV-1, causing a decrease in the ability of virions to infect cells and inhibiting viral attachment to cellular surfaces [36]. The HIV envelope protein gp41 plays a key role in the fusion between the viral envelope and the plasma membranes of target cells. The assembly of six-helix bundles (6HB) of gp41 is an indispensable step during fusion; subsequently, these 6HB predominantly govern the conformation of the fusion-active core. By blocking the 6HB formation of gp41, EGCG inhibits the membrane fusion of HIV-1 mediated by the envelope glycoprotein and thus blocks HIV-1 from entering cells [37]. The gp120 protein is another envelope glycoprotein of HIV that is essential for viral entry into cells, as it acts on the attachment to CD4 receptors on specific T-cell surfaces. EGCG can block gp120-CD4 binding through the preferential formation of the EGCG-CD4 complex, which results in an extreme reduction in the binding strength of gp120-CD4 [38]. Nuclear magnetic resonance spectroscopy, flow cytometry and molecular modeling from Williamson’s research group showed that the binding between EGCG and CD4 strongly reduced the formation of the gp120/CD4 complex. A binding site for EGCG was found on CD4 in the D1 domain, which is the gp120 binding pocket [39]. Using PBMCs, CD4 (+) T cells, and macrophages isolated from blood, Nance et al. reported that at physiological concentrations, EGCG weakened the binding activity of gp120 to CD4 by a dozen-fold [40]. 

EGCG attenuates neuronal damage mediated by HIV infection. HIV-associated dementia (HAD), previously referred to as AIDS dementia complex (ADC), is a condition caused by the action of HIV-1 infection on the central nervous system and leads to patients having difficulties with memory and learning. HAD is related to the activation of proinflammatory cytokines and neuropathology involving gp120 and Tat (a transactivating regulatory protein) of HIV. EGCG can mitigate neuron injury in the presence of IFN-gamma both in vitro and in vivo [41]. Using an HIV-1 Tat transgenic mouse model, Rrapo et al. demonstrated that EGCG could decrease the number of Tat-expressing astrocytes, mildly reduce activated microgliosis and enhance neuron survival [42]. In rodent cerebral cortical and hippocampal neuronal cultures, EGCG can function in the signaling pathway composed of brain-derived neurotrophic factor (BDNF) and its precursor proBDNF. Furthermore, EGCG balanced the increase in proapoptotic proBDNF and decrease in mature BDNF mediated by Tat [76].

Semen-derived enhancer of virus infection (SEVI) is a fibrillar structure and an important HIV-1 sexual infection factor that captures virions and guides them to their targets. EGCG can inhibit SEVI activity by complex formation and degradation [43,77]. Hartjen et al. confirmed and extended the findings of Hauber et al. using 47 fresh human semen samples [44]. They also observed a semen-independent inhibition of HIV-infectivity that the inhibition rate of HIV infectivity reached 88.5% post-treatment with 0.4 mM EGCG in the absence of semen. The findings above suggested that EGCG may be an alternative drug for preventing the sexual transmission of HIV.

### 3.2. EGCG Inhibits HCV

HCV is another major cause of chronic liver disease and can causes both acute and chronic infections. Timely antiviral therapy can cure more than 95% of persons with HCV infection, but access to diagnosis and treatment is currently low. According to the recommendations of the WHO, all patients with hepatitis C should be treated with sofosbuvir, daclatasvir and the sofosbuvir/ledipasvir combination based on direct-acting antivirals (DAA), except for a few specific groups of people in whom interferon-based regimens can still be used [78]. Although related researches are ongoing, there is currently no available effective vaccine. 

HCV is a *Flaviviridae* family member with a positive-sense single-stranded RNA genome and a small enveloped virus. In recent years, researchers have studied the anti-HCV activities of EGCG. Several independent research groups reported that EGCG was a potent inhibitor of the HCV entry pathway but had no effect on viral replication, viral RNA synthesis or virion secretion [10,46]. Moreover, Ciesek et al. found that EGCG can stop cell-to-cell transmission when the extracellular route of ingestion is blocked by overlaid agarose or incubated with neutralizing antibodies [10,45]. Besides EGCG, Calland’s group demonstrated that ECG and EGC had anti-HCV activities at an early step of the viral life cycle [10]. Chen et al. found EGCG not only suppressed HCV entry, but also inhibit viral RNA replication [47]. Recently, a new mechanism for the anti-HCV activity of GTCs was reported by Calland and co-workers [48]. Their results indicated that after EGCG treatment, an observed bulge was found on the viral particle and this kind of structural alteration did not result in destruction or aggregation of virons. [48]. The authors also identified delphinidin, a natural molecule with a similar structure to EGCG, as a new inhibitor of HCV infection that prevents HCV entry [48].

Using HCV JFH-1 infectious systems, Lin et al. reported that HCV replication was inhibited significantly by epicatechin isomers. They revealed that the mechanism of the anti-HCV activity of the epicatechin isomers most likely operates through the downregulation of COX-2 [49]. Moreover, these isomers suppressed inflammation by downregulating the expression of inflammatory factors such as tumor necrosis factor-alpha, interleukin-1 beta, inducible nitrite oxide synthase and COX-2 in viral protein-expressing hepatoma Huh-7 cells [49].Another interesting study evaluated the effect of oral doses of EGCG on cirrhotic patients with HCV and found that 400 mg of EGCG was safe and well tolerated; however, the cirrhosis of these patients did not obviously improve. Although the estimate was from limited samples, it provided guidance to researchers for further experiments or observations [79]. Results from Colpitts’s group demonstrated that EGCG interacted with surface proteins of dozens kind of virions, including HCV, and this interaction led to a failure of membrane fusion mediated by heparan sulfate- or sialic acid-containing glycans [28].

### 3.3. Inhibitory Effects of GTCs on Influenza Virus

Influenza virus includes the pathogens of flu outbreak in birds and many mammals including human, pig, horse, whales, seals, bat and so on. There are four types of influenza virus: A, B, C and D, which are categorized according to the antigenicity of the nucleocapsid protein. Influenza A virus (IAV) is a member of the family of Orthomyxoviridae and has a segmented single-stranded, negative-sense RNA genome. IAV is the main virus of the flu pandemic because of its high mutation rate. As early as 1949, Green et al. reported the antiviral activity of tea extracts against influenza virus [50]. The first serious discussion of the effects of EGCG against influenza A and B viruses was demonstrated in Madin-Darby canine kidney (MDCK) cells by Nakayama’s research group in 1993 [51]. They found that the infection of both IAV and influenza B virus (IBV) was inhibited by EGCG. Moreover, EGCG exerted agglutination effects on virions and prevented the virus from absorbing onto the cell surface [51]. Imanishi et al. further revealed that the anti-IV activity of green tea extracts that included EGCG possibly arose from its inhibitory effects on the acidification of endosomes and lysosomes [52]. 

To determine the relationship between the structure and activity of different GTCs, Song et al. evaluated their capabilities to inhibit the replication of viruses. They found that EGCG exerted more inhibitory effects than ECG and EGC on the activity of both viral neuraminidase and viral genomic RNA synthesis, suggesting the 3-galloyl group of the catechin skeleton was more important for antiviral activity than the 5’-OH in the trihydroxy benzyl moiety at the 2-position [53,54]. Some researchers explored the inhibitory effects of EGCG analogs, derivatives, and formulations on influenza virus [55,56,80]. Furuta et al. used deoxy-EGCG, a simplified analog of EGCG prepared by directly introducing a ketone group at C3, to show that the hydroxyl substituents on the A-ring of EGCG played a minor role in the anti-influenza virus activity [80]. Two interesting studies by Oxford’s group showed that QR-435, a natural extract from green tea, blocked transmission of IAV H3N2 and provided prophylaxis against H3N2. More interestingly, wearing masks containing QR-435 was able to prevent H3N2 infection [55,56]. Fatty acid monoester derivatives of EGCG, especially those with long alkyl chains, exhibited a sharply increased antiviral effect against IAV compared to natural EGCG [57]. The inhibitory effects of different nutrient mixtures of natural EGCG on influenza virus have also been demonstrated by different investigators [58,81,82]. Some researchers contributed to clinical trials of EGCG as an IAV restriction factor [59]. Moreover, one research group investigated the relationship between influenza virus infection and gargling tea catechin extract and demonstrated that GTCs significantly lowered the rate of influenza infection in 124 elderly residents aged more than 65 years [59]. In another randomized, double-blinded trial of 200 healthcare workers, consumption of capsules including GTCs for 5 months had a protective effect against IAV virus compared with the placebo group [60]. 

### 3.4. Effect of GTC on Some Arboviruses

Arbovirus refers to a group of viruses that are transmitted by insects, mostly commonly mosquitoes and ticks, sucking blood for nutrients or development [83]. The most common signs of infection with arboviruses are fever, headache, and general malaise. Severe patients present signs of encephalitis and hemorrhagic fever [84]. The infections caused by different arboviruses, including DENV, West Nile virus (WNV), Japanese encephalitis virus (JEV), tick-borne encephalitis virus (TBEV), and new outbreaks of ZIKV and CHIKV in Latin America, are now becoming serious health threats, especially in tropical and subtropical countries [83]. 

Recently, molecular docking methods proposed that EGCG can dock in the same binding domain of different E proteins from DENV, JEV and TBEV [14]. Furthermore, the amino acid residues associated with the DENV2 E protein were identified. CHIKV, which is an alphavirus, is transmitted by mosquitoes and causes chikungunya fever in humans. Weber et al. demonstrated the inhibitory effects of EGCG on CHIKV in vitro using a CHIKV-m Cherry-490 infection model. Moreover, they found that EGCG played a useful role in blocking CHIKV from entering the target cells and had a minor effect on CHIKV replication [15]. ZIKV, a member of the *Flaviviridae* family, has a single-stranded RNA genome and is spread by mosquitoes. Since its outbreak in Brazil in 2015, ZIKV has spread rapidly on a global scale [85]. EGCG showed antiviral activities against ZIKV according to a study from Carneiro et al. in 2015 [16]. Using Vero E6 cells and two strains of ZIKV, namely, ZIKVBR and MR766, they provided evidence for the vital anti-ZIKV function of EGCG. The associated mechanism was also explored. EGCG did not regulate the expression of cell receptors related to viral invasion. Therefore, it is speculated that there was a direct interaction between EGCG and the viral envelope, followed by destruction of the structure of ZIKV virions. 

### 3.5. Effect of GTCs on Human T-cell Lymphotropic Virus-1

Adult T-cell leukemia (ATL) is an aggressive T-cell malignancy caused by human T-cell leukemia virus type I (HTLV-1) infection. In the 30 years since HTLV-1 was discovered, of ATL tend to yield poor results, and little progress has been made in the cure rate [86]. Therefore, searching for new agents that target specific molecules and application of anti-HIV drugs needs to be encouraged [87]. Two groups independently contributed to the discovery of EGCG as an HTLV-1 restriction molecule and found that its inhibitory effect was achieved by suppressing HTLV-I pX and Tax gene expression [61,62]. 

### 3.6. Effect of GTCs on Rotaviruses and Enteroviruses

Rotaviruses and enteroviruses are viruses that can cause a series of intestinal symptoms. Enterovirus 71 (EV71) is the major pathogen of hand, foot and mouth disease. In addition, EV71 can lead to diarrhea, rashes and severe neurological disease. EV71 has done great harm to preschool children’s health in both developing and underdeveloped countries. As early as 1991, ECGC from green tea was reported to inhibit rotaviruses and enteroviruses in cultured rhesus monkey kidney cells by interfering with virus adsorption [63]. Ho et al. provided the first evidence for an anti-enterovirus function of GTCs [64]. The authors found that the production of infectious progeny virus of EV71 was reduced by 95% post-treatment with EGCG and gallocatechin gallate (GCG) and suggested that the inhibitory effects were related to the reduced reactive oxygen species (ROS) generation upon EGCG treatment [64].

### 3.7. Effect of GTCs on EBOV

The Ebola virus (EBOV) is among the most feared viruses and can cause Ebola hemorrhagic fever, a highly fatal disease. The recent WHO statistics showed that the 2014–2016 EBOV outbreak in West Africa had a high fatality rate of 28~75% [88]. Reid et al. identified a host chaperon protein, HSPS5, as an important target for therapies against EBOV infection and found that EGCG, as an inhibitor of HSPS5, reduced the production of new viruses via its action on HSPS5 [65]. 

### 3.8. Effect of GTCs on Viruses Infecting Other Animals

Fish and other animals not only are good dietary sources of protein, but also have great natural value and can provide substantial economic and scientific benefits. However, diseases caused by fish virus infections can effect heavy economic losses and influence the development of fish farming. EGCG has been suggested to be able to inhibit infections of some novirhabdo viruses, such as hemorrhagic septicemia virus (VHSV), hematopoietic necrosis virus (IHNV) and spring viremia carpvirus (SVCV), specifically because of its ability as a low-molecular-weight inhibitor of serine protease inhibitor gene transcripts 1 (SERPINe1) [66]. In 2016, the activity of GTCs against grass carp reovirus (GCRV) was demonstrated for the first time by Wang et al. [67]. The results showed that adhesion of GCRV virions to target cells was inhibited in a dose-dependent manner when the cells were treated with EGCG and crude extracts of green tea. It was inferred that the blocking effect of EGCG on GCRV attachment was due to the binding potential of GCRV particles to the laminin receptor (LamR) [67]. Porcine reproductive and respiratory syndrome (PRRS), an endemic pig disease, has caused great losses in the pig industry globally. Its pathogen is a peculiar and highly infectious virus, porcine reproductive and respiratory syndrome virus (PRRSV). Pregnant sows and piglets infected with PRRSV can develop severe reproductive deficiencies and respiratory symptoms, respectively. Using MARC-145 cells, Zhao et al. explored the anti-PRRSV activities of EGCG and EGCG palmitate in vitro. The authors found that the inhibitory effects of both EGCG and its palmitate on PRRSV were dose dependent and that EGCG palmitate showed a much stronger antiviral activity as a pretreatment compound than EGCG [13]. The activities of EGCG and EGCG-loaded chitosan microcapsules against murine norovirus were reported by Gomez-Mascaraque in 2016. The results demonstrated that the encapsulated EGCG was significantly more active than free EGCG [89]. 

## 4. Conclusions

Over the past few decades, GTCs, especially EGCG, have been recognized as multifunctional bioactive molecules responsible for antitumorigenic, anti-inflammatory, antioxidative, anti-proliferative, antibacterial, and antiviral effects [1]. In terms of the characteristics of different GTC structures, the differences in the functional properties of each catechin were associated with the number of hydroxyl groups on the B-ring and the presence of a galloyl group. Both pyrogallol and galloyl groups and the EGCG backbone itself play important roles in the different actions of different structures. Ultimately, the number and the positions of the hydroxyl groups in catechins are the most important factors influencing their activities. EGCG is thought to be the most potent component of the catechins because of its unique structural characteristics, namely, the presence of both pyrogallol and galloyl moieties [90,91]. These two groups are also essential for its antiviral activities, as both the hydroxyl group and galloyl group are necessary for the antiviral activities of GTCs. In addition, for different viruses or different viral protein markers, the phenolic hydroxyl group and the galloyl group likely have significantly different effects on the antiviral effects of GTCs [9,21,45,54]. In our research on anti-HBV activity, the phenolic hydroxyl groups of EGCG on the B-ring were found to play an important role in its inhibitory effect on HBsAg, whereas the galloyl group was more important for inhibiting HBeAg [9,21]. 

**Figure 2:**
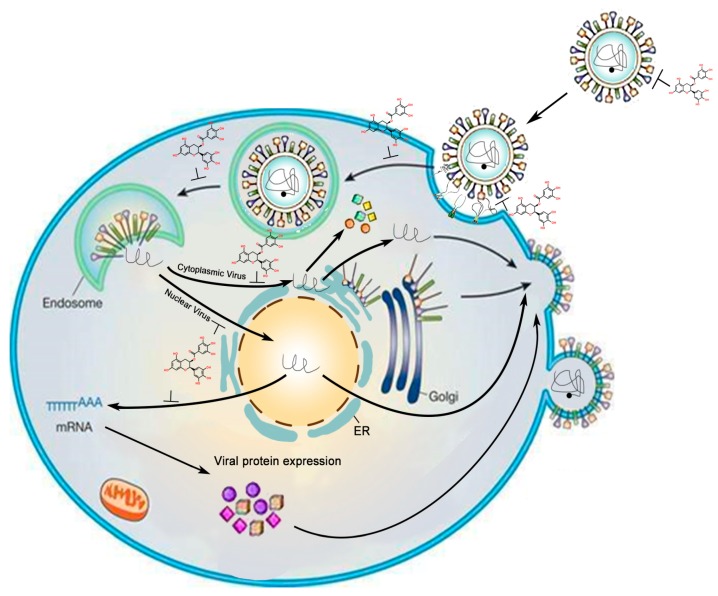
Inhibitory effects of EGCG against viruses at different stages of viral invasion. In this figure, to summarize many different viruses and models, according to the genome replication (omitting the type of nucleic acid), the viruses are divided into two types: nuclear virus and cytoplasmic virus. The symbol “⊥” indicates the sites of GTC functions its antiviral effects.

EGCG can be regarded as a nucleophilic reagent because the phenolic hydroxyl groups in the pyrogallol and galloyl moieties provide more lone pair electrons than other catechins. This type of structural feature allows EGCG to react or combine with different molecules under appropriate conditions. From the antiviral effects reported, whether the viral genome is DNA or RNA, GTCs seem to be able to function in different stages of infections of both nuclear viruses (the replication of viral genome occurs in the nucleus, such as all of DNA viruses and some RNA viruses) and cytoplasmic RNA viruses (Figure 2). The inhibitory effects of EGCG on multiple viruses indicate that this compound is a potential alternative agent for viral diseases. 

The many previous investigations into the mechanism of action of EGCG indicate that the compound binds strongly to many molecules in cells, especially proteins, and then affects their original activities and functions [73,92,93,94,95,96]. By interacting with the virion surface or cell surface receptors, EGCG can interfere with the interaction between the virions and the host cells. EGCG plays an important role in regulating the microenvironment of endosomes and lysosomes, the acidification of which is crucial for viral invasion. Viral genome replication or viral protein expression can also be suppressed because of the inactivation of viral replicases or regulation of host factors. However, all the existing achievements have demonstrated the inhibitory effects of EGCG on the stages between virus attachment and genome synthesis. To date, few antiviral effects on viral protein translation and virus assembly and budding have been reported. 

In the future, studies on the antiviral molecular mechanism, especially those on the molecules that bind with EGCG, will not only provide new insights into antiviral therapy but also contribute to the discovery of new targets. However, at least for now, the GTCs do not make good drugs, mainly because of their poor pharmacokinetic/pharmacodynamic (PK/PD) properties. Because of the unstable special structure and complexity of human physiological metabolism and cells, EGCG is easily oxidized or converted into other structural forms before entering its action targets. Therefore, structural transformation and modification and analog synthesis may subsequently become research emphases in EGCG studies.

Scientists have described some derivatives through replacing the different hydrogens on the rings of A, B and D of EGCG structure, such as methylated [97,98], acylated [99,100,101,102], esterified [103,104], and glycosylated [105,106,107] EGCG, in research relating to other activities of GTCs. From the existing research data, it seems that the modifications on the rings of B and D, especially D show more effectivity. 

**Figure 3:**
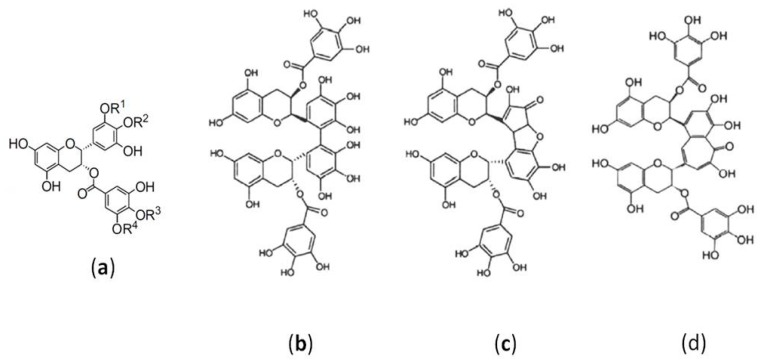
The structurally modified EGCG or dimers with with stronger effects against virus. (**a**) EGCG-monopalmitate derivatives composed of four regioisomers in different proportions of 1, 2, 3 and 4 against IAV and HSV-1. The EGCG dimers theasinensin A (**b**), P2 (**c**), and theaflavin-3, 3-digallate (**d**) inactivating HSV-1 and HSV-2 more effectively than monomer.

In recent years, this area of exploration has diversified into embedded forms of EGCG. For example, encapsulated nanosomes [108,109,110,111] offer new insights to researchers in this field. Several prospective studies have provided interesting findings on the antiviral activities of structurally modified EGCG or dimers (Figure 3a–d) [25,27,57]. Results showed EGCG-monopalmitate derivatives composed of four regioisomers in different proportions of 1, 2, 3 and 4 had stronger effects against IAV [57] and HSV-1[27]. A case study demonstrated that EGCG-stearate could effectively prevent and improve HSV-induced symptoms [26]. The findings mentioned in this review have stimulated the interest of many researchers and promoted progress in this field. Despite the studies mentioned above, there is still a lack of pharmacological and PK/PD studies. Appropriate PK/PD modeling and simulation can indicate some potential approaches to help overcome the limitations of GTCs. In addition, most of the research data summarized here were based on in vitro experiments [112]. To investigate the effects and mechanisms of GTC activity against viruses, conducting related studies in vivo or in animals is crucial. Regarding the discovery of antiviral drugs, this is a promising area and will become an important development trend in the future because many more studies will be needed before viral infection and diseases can be fully understood.

## Figures and Tables

**Table 1:** Inhibitory effects of viruses by green tea catechins.

Virus	Family	Genome	Effect	Effective Dosage or Concentration, Year (Reference)
HBV	*Hepadnaviridae*	Partially double-stranded and circular DNA	Inhibition of HBV RNA, DNA, and cccDNA synthesis and antigen expression EGCG targets replicative intermediates of DNA synthesis Interference with transcription of the HBV core promoter Inhibition of different genotypes of HBV entry into cells Reduction of HBV replication by opposing HBV-induced incomplete autophagy	EC_50_ (GTE, HBsAg) = 5.02 mg/mL, on, EC_50_ (GTE, HBeAg) = 5.681 mg/mL, EC_50_ (GTE, HBV DNA) = 19.81 mg/mL, 2008 [9]; EGCG (25, 50, 100 μM), 2011 [20] EGCG (25, 50, 100 μM), 2016 [21] EGCG (10, 20, 50 μM), 2014 [22] EGCG (25, 50 μM), 2015 [23]
HSV	*Herpesviridae*	Double-stranded linear DNA	Strong anti-HSV activity Inactivating clinical isolates of HSV by destruction of the virion structure Improved anti-HSV effectiveness of modified EGCG Inhibition of HSV-1 attachment by interacting with the virion surface	EC_50 (EGCG,EGC,EC) HSV-1_ = 2.5 µM, EC_50 (ECG) HSV-1_ = 4 µM, EC_50 (EC) HSV-2_ = 35 µM, EC_50 (ECG) HSV-2_ = 63 µM, 2005 [11] IC_99 (EGCG) HSV-1_ = 16–49 µM, IC_99 (EGCG) HSV-2_ = 12.5 µM, IC_99 (EGCG) Lab strain HSV-1_ = 72.3 µM, 2008 [24]; Digallate dimers of EGCG (100 μM), 2011 [25]; EGCG-stearate in 100% glycerin USP, 2012 [26], palmitoyl-EGCG, 2013 [27] EGCG (0.01–200 µM), 2014 [28]
EBV	*Herpesviridae*	Double-stranded linear DNA	Reduction of EBV lytic protein expression Interference with transduction of the AP-1 signal pathway Decreasing binding activity of DNA and nuclear antigen 1 and blocking EBV lysis by down regulating RNA synthesis of viral immediate-early genes Blocking EBV spontaneous lytic infection by interfering with the MEK/ERK1/2 and PI3-K/Akt pathways	EGCG > 50 µM, 2003 [29] EGCG (25, 50, 100, 200 µM), 2004 [29] EGCG (10, 30, 50 µM), 2012 [30] IC_50 (EGCG)_ = 20 µM, 2013 [31]
Adenovirus	*Adenoviridae*	Double-stranded linear DNA	Inhibition of viral titers of adenovirus and inactivation of purified adenovirions and adenain Inhibition of viral attachment by interacting with virion surface proteins	IC_50 (EGCG,Effect on infectious virus production)_ = 25 µM, IC_50 (EGCG,Inactivation of adenovirus)_ = 250 µM, IC_50 (EGCG, Effect on adenain)_ = 109 µM, 2003 [32] EGCG (0.01–200 µM), 2014 [28]
HIV	*Retroviridae*	+ssRNA	Inhibition of HIV RT Inhibition of viral entry into target cells by interfering with the interaction of receptors with the HIV envelope Inhibition of p24 antigen production Attenuation of neuronal damage mediated by HIV infection Counteraction of semen-mediated enhancement of HIV infection	EC_50 (EGC, EGCG)_ = 21.8–65.3 μM (0.01–0.02 mg/mL), 1990[33]; EC_50 (EGC)_ = 25.5 μM (7.8 mg/L), EC_50 (ECG)_ = 0.72 μM (0.32 mg/L), EC_50 (EGCG)_ = 1.48 μM (0.68 mg/L), 1994 [34]; EC_50 (EGCG)_ = 1.6~2.0 μM, 2011 [35] EGCG (10~100 μM, 2002 [36]; IC_50 (EGCG)_ = 3.44, IC_50 (GCG)_ = 2.45, 2005 [37]; IC_50 (EGCG)_ ≈ 100 μM, 2006 [38]; EGCG (0.2 μM), 2006 [39]; IC_50_ ≈ 4.5 μM, 2009 [40] EGCG (20 μM in vitro; 50 mg/kg, mouse model), 2006 [41]; EGCG (300 mg/kg/day, mouse model), 2009 [42] EGCG (1~20 mM), 2009 [43]; EGCG (0.4 mM), 2012 [44]
HCV	*Flaviviridae*	+ssRNA	Inhibition of the HCV entry pathway, prevention of cell-to-cell transmission Supression of HCV RNA replication steps Impairment of viral attachment by altering viral particle structure Interference with HCV replication by down regulating a COX-2 inhibitor Targeting the HCV virion to prevent attachment to heparan sulfate)	IC_50 (EGCG, Cell-culture–derived HCV entry)_ = 2.5 μg/mL, IC_50 (EGCG, binding of HCV to cells, with or without)_ = 9.7 μg/mL or 17.2 μg/mL, 2011 [45]; EGCG (0.625~10 μM), 2012 [46]; EC_50 (EGCG)_ = 17.9 μM, 2012 [47]; IC_50 (EGCG)_ = 10.6 ± 2.9 μM, IC_50 (delphinidin)_ = 3.7 ± 0.8 μM, 2015 [48] EC isomers (25, 50, 75 μM), 2013 [49] EGCG (0.01–200 µM), 2014 [28]
Influenza virus	*Orthomyxoviridae*	−ssRNA	Antiviral activity of tea extracts against influenza virus Infectivity reduction of IAV and IBV by preventing viral absorption to cell surface and inhibiting acidification of endosomes and lysosomes Activity reduction of viral neuraminidase and RNA synthesis of viral genome Inhibitory effects of influenza virus by EGCG analogs, derivatives and compounds Inhibitory effects of different nutrient mixtures of natural EGCG Clinical trials of EGCG as an influenza virus restriction factor	1949 [50] EGCG (1–16 μM), 1993 [51]; GTE (1:20, 1:40, 1:80 dilutions), EGC (400 µg/mL), 2002 [52] EC_50 (EGCG)_ = 22–28 μM, EC_50 (ECG)_ = 22–40 μM, EC_50 (EGC)_ = 309–318 μM, 2005 [53]; IC_50 (GTC tested, IAV)_ = 16.2–56.5 µg/mL, IC_50 (GTC tested, IBV)_ = 9.0–49.7 µg/mL, 2014 [54] QR-435, 2007 [55,56]; Fatty acid (3-O-acylcatechins), 2008 [57] 2007 [58], 2008 [58] Gargling with tea catechin extracts solution (200 µg/mL catechins, ECGC composes 60% of catechins), 2006 [59], GTC (378 mg/day), 2011 [60]
DENV, JEV,TBEV ZIKV	*Flaviviridae*	+ssRNA	Docking into the binding pocket of E protein Destruction of the virus particle by interacting with the lipid envelope	2016 [14] EC_50 (EGCG)_ = 21.4 µM, 2016 [16]
CHIKV	*Togaviridae*	+ssRNA	Blocking CHIKV entry into target cells	IC_50 (EGCG)_ = 14.3 µM (6.54 µg/mL), 2015 [15]
HTLV-1	*Retroviridae*	+ssRNA	Suppressing HTLV-I pX and Tax gene expression	EGCG or GTP (6.5–60 µM, 3–27 µg/mL), 2000 [61]; EGCG (25, 50, 75 µM) effective in C91-PL cells, EGCG (125, 225, 325 µM) effective in HuT-102 cells, 2014 [62]
Rotavirus	*Reoviridae*	dsRNA	Interference with virus adsorption	1991 [63]
Enterovirus EV71	*Picornaviridae*	+ssRNA	Interference with virus adsorption Inhibition of production of progeny virus by reducing ROS generation	1991 [63] EGCG (25 μM), 2009 [64]
EBOV	*Filoviridae*	−ssRNA	As an inhibitor of HSPS5, EGCG reduced the production of new viruses via its action on HSPS5	EGCG (10–100 μM), [65]
PRRSV	*Arteriviridae*	+ssRNA	Inhibition of viral adsorption and cell intrusion of PRRSV by EGCG palmitate	10TCID_50_: EC_50 (EGCG,pretreated)_ = 8.53 µM, EC_50 (EGCGpalmitate, pretreated)_ = 0.58 µM, EC_50 (EGCG,post-treated)_ = 9.18 µM, EC_50 (EGCGpalmitate, post-treated)_ = 0.68 µM, 2014 [13]
VHSV, IHNV, SVCV	*Rhabdoviridae*	−ssRNA	Reduction of rhabdovirus infections by inhibitingSERPINe1 activity	EGCG (10, 100 µM), 2015 [66]
GCRV	*Reoviridae*	dsRNA	Interference with interaction of GCRV particles with laminin receptor	IC_80 (EGCG)_ = 21.8 µM (10 µg/mL), 2016 [67]

**Note:** dsRNA, double-stranded RNA; +ssRNA, positive-sense, single-stranded RNA; −ssRNA, negative-sense, single-stranded RNA; EC_50_, 50% effective concentration; IC_99_, 99% effective concentration; IC_50_, half maximal inhibitory concentration; IC_80_, 80% maximal inhibitory concentration; TCID_50_, Tissue culture infective dose; EGCG (concentration), effective concentration used in research.
